# Large-Deformation Image Registration of CT-TEE for Surgical Navigation of Congenital Heart Disease

**DOI:** 10.1155/2018/4687376

**Published:** 2018-07-30

**Authors:** Shuiping Gou, Linlin Chen, Yu Gu, Liyu Huang, Meiping Huang, Jian Zhuang

**Affiliations:** ^1^Key Laboratory of Intelligent Perception and Image Understanding of Ministry of Education of China, Xidian University, Xi'an 710071, China; ^2^Department of Biological Science and Engineering, Xidian University, Xi'an 710071, China; ^3^Catheterization Lab, Guangdong Cardiovascular Institute, Guangdong Provincial Key Laboratory of South China Structural Heart Disease, Guangdong General Hospital, Guangdong Academy of Medical Sciences, Guangzhou 510000, China; ^4^Department of Cardiac Surgery, Guangdong Cardiovascular Institute, Guangdong Provincial Key Laboratory of South China Structural Heart Disease, Guangdong General Hospital, Guangdong Academy of Medical Sciences, Guangzhou 510000, China

## Abstract

The surgical treatment of congenital heart disease requires navigational assistance with transesophageal echocardiography (TEE); however, TEE images are often difficult to interpret and provide very limited anatomical information. Registering preoperative CT images to intraoperative TEE images provides surgeons with richer and more useful anatomical information. Yet, CT and TEE images differ substantially in terms of scale and geometry. In the present research, we propose a novel method for the registration of CT and TEE images for navigation during surgical repair of large defects in patients with congenital heart disease. Valve data was used for the coarse registration to determine the basic location. This was followed by the use of an enhanced probability model map to overcome gray-level differences between the two imaging modalities. Finally, the rapid optimization of mutual information was achieved by migrating parameters. This method was tested on a dataset of 240 images from 12 infant, children (≤ 3 years old), and adult patients with congenital heart disease. Compared to the “bronze standard” registration, the proposed method was more accurate with an average Dice coefficient of 0.91 and an average root mean square of target registration error of 1.2655 mm.

## 1. Introduction

Congenital heart disease accounts for 28% of all congenital malformations. In China, the incidence of congenital heart disease is 0.4–1% among infants with 150000–200000 newborns annually. Imaging of congenital heart disease includes cardiovascular magnetic resonance (CMR), computed tomography (CT), transesophageal echocardiography (TEE), and other modalities. CMR provides comprehensive data on the mechanism of congenital heart disease without the need for radiography or invasive procedures; however, it requires subjects to remain stationary for 15–60 min, which can be challenging in infants and children. Additionally, the high cost of CMR limits its clinical application. High-resolution CT can be rapidly imaging and provide a wealth of anatomical data; however, the equipment of it is not easy to move and cannot be used in surgery. TEE allows a continuous visualization of the movement of the visceral organ without trauma and the observation of the heartbeat in real time, which is convenient to use in surgery; thus, it can be useful for navigation during the surgery. However, its images provide very limited data on cardiac anatomy, it often requires the use of another imaging modality, and there is no established method for accurately registering anatomical structures to TEE images. Each type of imaging modality has advantages and disadvantages, so image fusion is necessary.

Image registration is the premise of image fusion, which has significant value in the fields of remote sensing data analysis, computer vision, and image processing. Feature point-based registration is one of the most commonly used image registration methods. Typical feature points include corner points, line intersections, centroids of closed curves, maximum curvature points on the contour, local curvature discontinuities detected by Gabor wavelet [1], and local maximum points of wavelet transform [2]. Feature points can be extracted manually or automatically. There are many methods to automatically extract feature points. Feng et al. [3] proposed a 3D feature point extraction using a neural network. Cheng et al. [4] adapted multilevel feature point extraction into a Naïve Bayesian classification framework. Chen et al. [5] presented a novel method of 3D XYT feature point extraction. Dán et al. [6] extracted feature points by characterizing of SURF and BRISK Interest Point Distribution. Wang et al. [7] introduced a multiscale and hierarchical feature extraction method. The feature point-based registration can greatly reduce the computational complexity of the matching process and has better adaptability to the change of gray level, image deformation, and occlusion. However, when the image modalities differ greatly, the algorithm based on automatically selecting the feature points has poor registration accuracy and may even result in completely failed results. Therefore, typically, the coarse registration was implemented by selecting feature points, and then the fine registration was carried out based on the correlation matching techniques.

Similarity measures are critical for the correlation matching techniques. The sum of squared differences (SSD), sum of absolute differences (SAD) [8], normalized cross correlation (NCC) [9], and mutual information (MI) [10] are commonly used similarity measures. SSD and SAD are particularly sensitive to the gray scale transformation of the input image and are often used for monomodality image registration. The sensitivity of NCC to abnormal points affects the registration. MI was previously proposed by Viola et al. [10] and Maes et al. [11] as an insensitive method to differences in intensities between images and as a method that does not require feature extraction or segmentation that has cultivated attention recently. It is widely used for the registration of multimodal images; however, it is not a universal method. The registration function of MI can be ambiguous and can contain local extrema [12]. Therefore, it is common to combine MI with other imaging features with the aim of improving registration and having an accurate correspondence on difficult registration issues. Gong et al. [13] combined MI with SIFT to realized an automatic image registration for remote sensing images. Woo et al. [14] demonstrated a multimodal Registration via Mutual Information Incorporating Geometric and Spatial Context. Atli et al. [15] combined contrast enhancement techniques with MI-based image registration. Legg et al. [16] used the feature neighbourhood mutual information for multimodal image registration. Pradhan et al. [17] incorporated utility measure into enhanced mutual information as a weighted information to the joint histogram of the images.

Most of the aforementioned similarity measures use joint probability models of single pixels or voxels. By randomly changing the location of pixels or voxels on images and evaluating the statistical criterion, the same similarity is obtained [18]. This means that when spatial information between pixels or voxels is ignored, registration may fail because the matching criterion is not able to quantify the difference between the two images. Using spatial distributions of pixels or voxels rather than single pixels or voxels, Penney et al. [19] successfully aligned ultrasound images and MR images of the liver by registering vessel probability images. This technique used a probability map to enhance corresponding features and depress those that were noncorresponding between the images. However, the generation of a probability density functions as proposed by the authors required a large amount of training data that were different from the actual test data. This approach cannot be easily applied to cardiac CT and TEE image registration. Additionally, the probability map proposed by Penney et al. yielded evident deviations, which may not be acceptable for the registration of cardiac CT and TEE images. In this study, an enhanced probability map model is proposed to overcome the shortage of training data and the deviation of the aforementioned probability map.

The difficulties in cardiac CT-TEE registration lie in the displacement caused by cardiac motion, as well as the considerable differences in the distribution of gray levels, structural features, and edge features. Determining methods to overcome the above difficulties is the key issue in cardiac CT-TEE registration. At present, there are few studies on cardiac CT/MR-TEE registration. D. S. Cho et al. [20] and C. A. Linte et al. [21] adopted a landmark-based registration method to reduce image displacement and modal differences, both of which relied on custom-developed equipment to extract landmarks. P. Lang et al. [22] focused only on the cardiac CT-TEE registration of the aortic root. F. Li et al. [23] performed a registration using a two-stage approach. In the first step, ICP algorithm was performed on the basis of semiautomatic segmentation. In the second step, sample points were extracted by setting intensity thresholds; then, these sample points were used to perform a finer registration step based on MI. This method involves setting different intensity thresholds manually according to different images.

Most of the existing cardiac CT-TEE registrations require the assistance of hardware devices and manual intervention. Some studies only focused on the cardiac registration of certain special sections. In this paper, a registration method is proposed for all cardiac sections, which can minimize manual intervention and does not require additional assistance of hardware equipment. Considering that heart valves are clearly visible on TEE images and are also easily located on CT images, this paper introduced the spatial positions of valves as a priori information. Then, coarse registration was performed based on valve positions to narrow the position difference in CT-TEE registration. To overcome the limitation of the probability map proposed by Penney et al., an enhanced probability map model was proposed by generating the probability map based on the enhancement of regions of interest. It can better characterize the spatial distribution of gray levels and can directly generate the probability density function by using images for subsequent registration. Moreover, we used the transformation parameters from coarse registration as the initial values for the optimization algorithm in the final registration to prevent the optimization algorithm from falling into a local optimum.

## 2. Materials and Methods

### 2.1. Method Overview

Registration was performed using valves as a landmark. Probability map-based MI (VPMMI) consisted of basic registration using cardiac valves as input data, while final registration was based on the probability map. This method was named VPMMI. Considering the demand for valve positions in actual application in this research context, the spatial locations of valves were introduced as a priori information to improve the effectiveness of CT and TEE image registration. With the a priori information of valve, we performed a simple and quick coarse registration. Errors in determining the initial values may cause an optimization algorithm to fall into a local optimum. To avoid this, we used the transformation parameters from coarse registration as the initial values for the optimization algorithm in the next phase of registration. Finally, in order to further improve the registration accuracy, the enhanced probability map was used to perform a finer registration step, which can overcome the shortage of training data. To sum up, our method is as follows.

Cardiac CT *I*_*R*_(*x*) was used as the reference image, while TEE *I*_*F*_(*x*) was used as the floating image, where *x* denoted a point on the 2 image spaces *Ω*_*R*_ and *Ω*_*F*_. The goal of image registration was to find a suitable transformation *T*:*Ω*_*F*_ → *Ω*_*R*_ in order to maximize the similarity *S*(*T*; *I*_*F*_; *I*_*R*_) between *I*_*R*_(*x*) and T(*I*_*F*_(*x*)). In this paper, transformation *T* comprised *T*_*basic*_ and *T*_*final*_. *T*_*basic*_ was the transformation obtained from basic registration that was used as the initial parameter for the optimization algorithm for the final registration. *T*_*final*_ transformed positions from the floating to the reference images.

To achieve the aforementioned goals we performed the steps shown in Algorithm 1.

**Figure 1:**
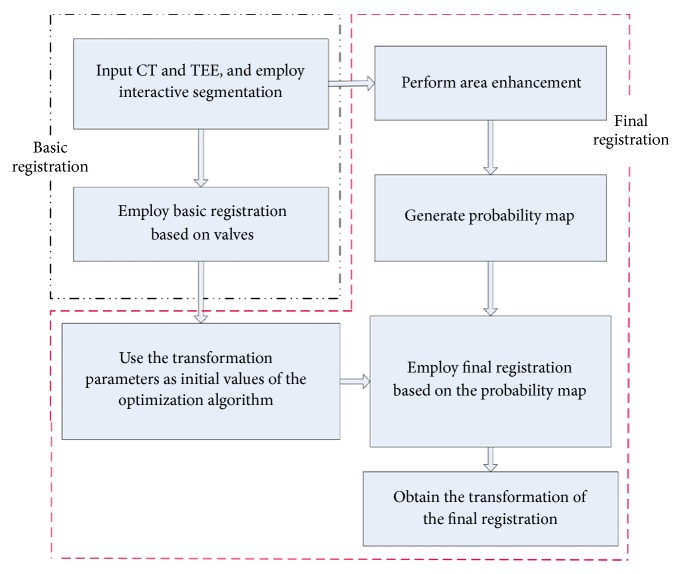
Flowchart of the proposed algorithm. CT, computed tomography; TEE, transesophageal echocardiography.

A flowchart of the proposed algorithm is shown in Figure 1.

### 2.2. Basic Registration 

#### 2.2.1. Interactive Segmentation

**Figure 2:**
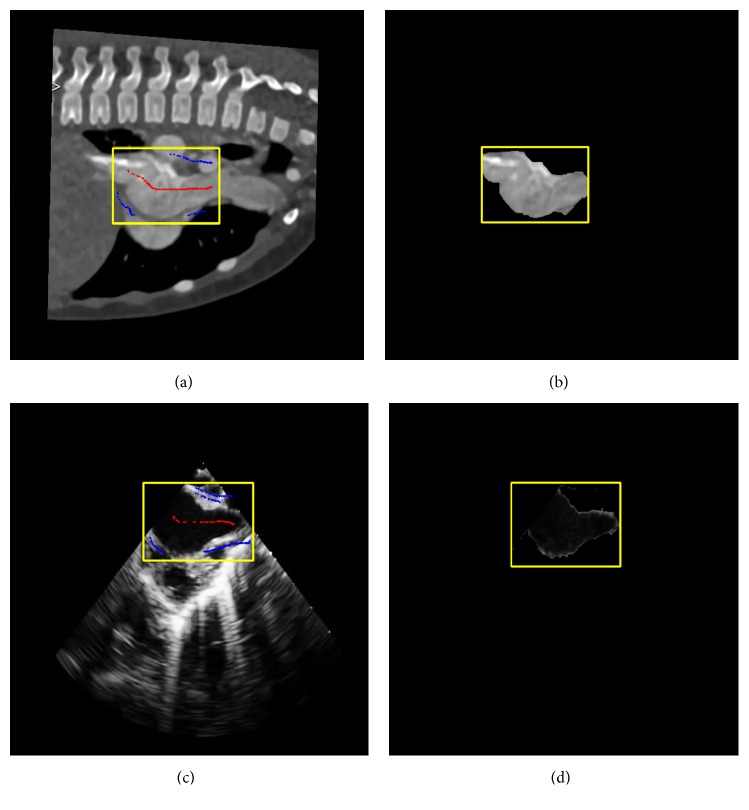
The process of interactive segmentation. (a) Interactive segmentation of a CT image. (b) The result of CT image segmentation. (c) Interactive segmentation of a TEE image. (d) The result of TEE image segmentation. Note: during interactive segmentation, the user selected a region of interest (yellow box) and marked the target (red dots) and the background areas (blue dots). CT, computed tomography; TEE, transesophageal echocardiography.

User interactions were adopted to introduce feature points and generate subsequent probability maps. We employed the grab-cut algorithm [24] for interactive segmentations, which is an image segmentation algorithm used to minimize energy function for boundary detection. In order to rapidly obtain segmentation images, the physician manually selected ROIs and marked the target and background areas on CT and TEE images.  Figure 2 shows the interactive segmentation of CT and TEE images.

#### 2.2.2. Introducing Heart Valve Locations as A Priori Information

Heart valves play an important role in cardiac circulation. Atrioventricular (between atria and ventricles) and semilunar valves (in arteries leaving the heart) help prevent the backward flow of blood. They can be clearly visualized on TEE and their approximate locations can be determined on CT scans by professionals. Therefore, it is reasonable to use valve positions as a priori information.

During interactive segmentation, three endpoints or midpoints coordinates *x*_1*r*_ = (*i*_1*r*_, *j*_1*r*_), *x*_2*r*_ = (*i*_2*r*_, *j*_2*r*_), *x*_3*r*_ = (*i*_3*r*_, *j*_3*r*_) of heart valves on the CT *I*_*R*_(*x*) image were determined as three feature points on CT. Similarly, three feature points *x*_1*f*_ = (*i*_1*f*_, *j*_1*f*_), *x*_2*f*_ = (*i*_2*f*_, *j*_2*f*_), *x*_3*f*_ = (*i*_3*f*_, *j*_3*f*_) were determined on the TEE image *I*_*F*_(*x*).

#### 2.2.3. Basic Registration Based on Feature Points

Using the three CT feature points *x*_1*r*_ = (*i*_1*r*_, *j*_1*r*_), *x*_2*r*_ = (*i*_2*r*_, *j*_2*r*_), *x*_3*r*_ = (*i*_3*r*_, *j*_3*r*_) and the three TEE feature points *x*_1*f*_ = (*i*_1*f*_, *j*_1*f*_), *x*_2*f*_ = (*i*_2*f*_, *j*_2*f*_), *x*_3*f*_ = (*i*_3*f*_, *j*_3*f*_), the coordinate transformation matrix *T*_*basic*_ of the basic registration below was obtained.

(1) $$\begin{array}{c} \left[\begin{array}{c} x_{1r}\\ x_{2r}\\ x_{3r} \end{array}\right]=T_{basic}\times \left[\begin{array}{c} x_{1f}\\ x_{2f}\\ x_{3f} \end{array}\right] \end{array}$$

Based on *T*_*basic*_, the results of basic registration *S*_*basic*_(*x*) were obtained by performing an affine transformation and a cubic interpolation to the floating image *I*_*F*_(*x*).

### 2.3. Final Registration

#### 2.3.1. Enhancement of CT and TEE Images

**Figure 3:**
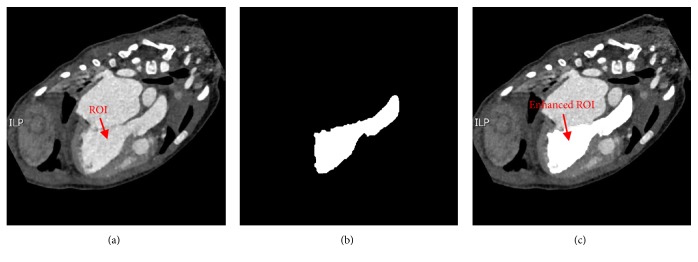
The process of region enhancement. (a) Original CT image *I*_*R*_(*x*). (b) Enhancement matrix *V*_*R*_(*x*). (c) Enhanced CT image. CT, computed tomography; ROI, region of interest.

In order to generate an improved probability map for good performance in the subsequent registration, we performed region enhancement to improve the contrasts between the foreground and the background. First, we set an enhancement matrix *V*_*R*_(*x*) for the CT image *I*_*R*_(*x*). *V*_*R*_(*x*) was constructed such that the size of *V*_*R*_(*x*) was the same as the size of the CT image *I*_*R*_(*x*) and the pixels corresponding to the coordinates of the ROI on *I*_*R*_(*x*) were set to a number N (N set to 80 in our experiment; this value was based on the average ROI intensity and empirical experiments) and 0 for the remaining pixels. The process of region enhancement is shown in Figure 3.

Subsequently, we enhanced *I*_*R*_(*x*) and its segmentation *G*_*R*_(*x*) to obtain the enhanced CT image *I*_*Rh*_(*x*) and enhanced segmentation *G*_*Rh*_(*x*):

(2) IRhx=IRx+VRx

(3) GRhx=GRx+VRx

Similarly, we generated a TEE image enhancement matrix *V*_*F*_(*x*). *V*_*F*_(*x*) was constructed such that the size of *V*_*F*_(*x*) was the same as the size of the TEE image *I*_*R*_(*x*) and the pixels corresponding to the coordinates of the ROI in *I*_*R*_(*x*) were set to a number M (M was 100 in our experiment; it was an empirical value based on the average ROI intensity of many experiments) and 0 for the remaining pixels. Finally, *I*_*F*_(*x*) and its segmentation *G*_*F*_(*x*) were enhanced to obtain the enhanced TEE image *I*_*Fh*_(*x*) and enhanced segmentation *G*_*Fh*_(*x*):

(4) IFhx=IFx+VFx

(5) GFhx=GFx+VFx

It is worth mentioning that although we strengthened the pixel intensity on this step, we did not change the spatial relationship between pixels. Therefore, the subsequent probability map model was not affected.

#### 2.3.2. Probability Map Generation

Instead of using pixel intensity, the probability map described image information, i.e., the probability that a pixel was located inside the ROI. Probability mapping enhances relevant features and depresses irrelevant features between two images. Based on the region-enhanced CT image *I*_*Rh*_(*x*) and its enhanced segmentation *G*_*Rh*_(*x*), the probability density function of CT *f*_*R*_(*i*) was generated as follows:

(6) $$\begin{array}{c} f_{R}\left(\;i\;\right)=\frac{Number\;\;of\;\;G_{Rh}\left(x\right)\;\;pixels\;\;of\;\;intensity\;\;i}{Number\;\;of\;\;I_{Rh}\left(x\right)\;\;pixels\;\;of\;\;intensity\;\;i}\% \end{array}$$

The probability density function *f*_*R*_(*i*) corresponds to a retrieval table that outputs a probability that a given pixel is located within the ROI when a pixel value is used as an input. In the same way, we derived the probability density function of TEE *f*_*F*_(*i*) as follows:

(7) $$\begin{array}{c} f_{F}\left(\;i\;\right)=\frac{Number\;\;of\;\;G_{Fh}\left(x\right)\;\;pixels\;\;of\;\;intensity\;\;i}{Number\;\;of\;\;I_{Fh}\left(x\right)\;\;pixels\;\;of\;\;intensity\;\;i}\% \end{array}$$

**Figure 4:**
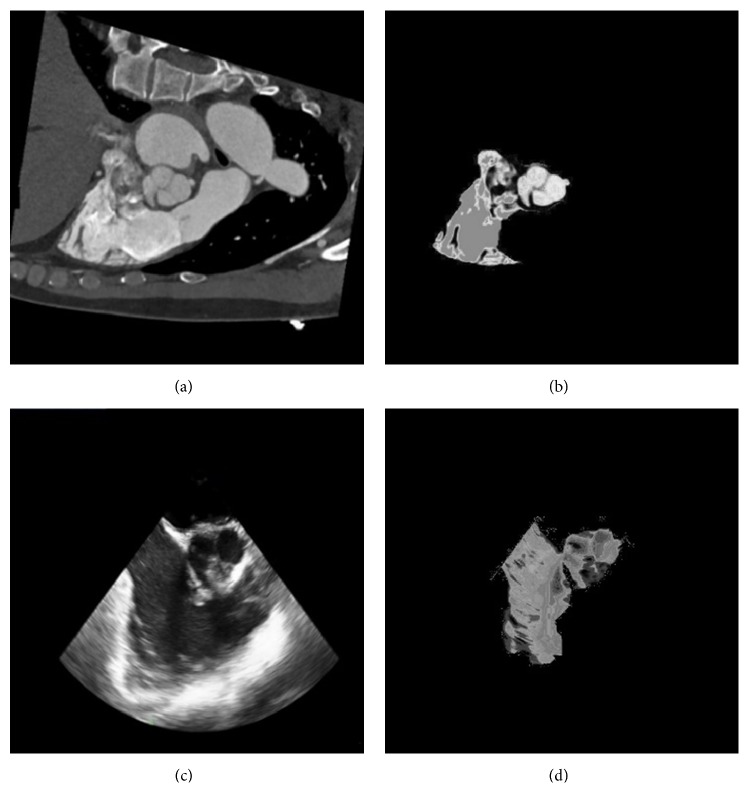
CT and TEE probability maps. (a) The original CT image. (b) The CT probability map. (c) The original TEE image. (d) The TEE probability map. CT, computed tomography; TEE, transesophageal electrocardiography.

Subsequently, we used the region-enhanced CT image *I*_*Rh*_(*x*) as the input of *f*_*R*_(*i*) to develop the probability map *P*_*R*_(*x*): *P*_*R*_(*x*) = *f*_*R*_(*i*), *i* ∈ *I*_*Rh*_(*x*). Similarly, using the region-enhanced TEE image *I*_*Fh*_(*x*) as the input of *f*_*F*_(*i*), we developed the probability map *P*_*F*_(*x*):*P*_*F*_(*x*) = *f*_*F*_(*i*), *i* ∈ *I*_*Fh*_(*x*). CT and TEE probability maps are shown in Figure 4.

#### 2.3.3. Final Registration Based on the Maximum Mutual Information of the Probability Map

We used Powell's algorithm as an optimization algorithm with initial parameters obtained from transforming matrix *T*_*basic*_. The transform matrix of the final registration *T*_*final*_ was yielded when the normalized mutual information (NMI) between *P*_*R*_(*x*) and *P*_*F*_(*x*) was maximized through Powell's algorithm. Afterward, the final registration result *S*_*final*_(*x*) was derived as follows: *S*_*final*_(*x*) = *T*_*final*_ × *I*_*F*_(*x*).

## 3. Results and Discussion

The proposed algorithm was tested on a dataset consisting of 240 images from 12 patients with congenital heart disease (identified as Patient 1–Patient 12). Patients 11 and 12 were adults and the remaining 10 patients were infants and young children ≤ 3 years of age. Each patient's data included CT images and corresponding TEE images. Due to the difficulty of data acquisition, TEE images for each patient only included 22 standard sections of the heart. The sizes of all CT images were 1024 × 1024 and the sizes of all TEE images were 600 × 800.

All tests were executed on an Intel i5-6500 (Intel Corp., Santa Clara, CA) CPU 3.2 GHz, and the code was written using MATLAB R2015b (MathWorks, Natick, MA) development environment.

### 3.1. Comparison Algorithms

**Figure 5:**
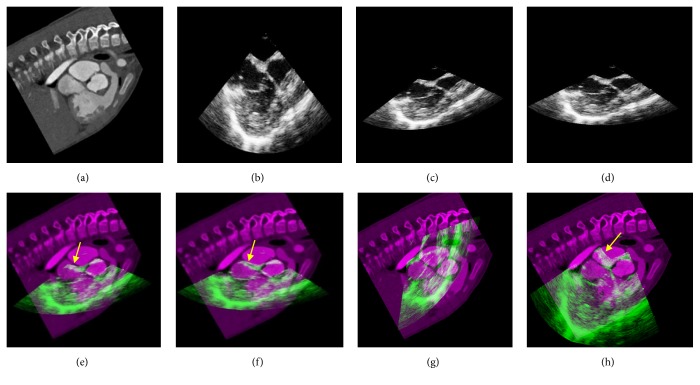
Registration process for a set of sections from an infant patient (Patient 8 in Table 1). (a) The original CT image. (b) The original TEE image. (c) The result of basic registration. (d) The result of final registration. (e) The fusion result based on the basic registration. (f) The fusion result based on the final registration. (g) The fusion result obtained from the comparison algorithm VMI. (h) The fusion result obtained from the comparison algorithm ICPMI. Note: the yellow arrows in (e), (f), and (h) indicate the alignment of the heart valves. CT, computed tomography; TEE, transesophageal electrocardiography; VMI and ICPMI, comparison algorithms.

**Figure 6:**
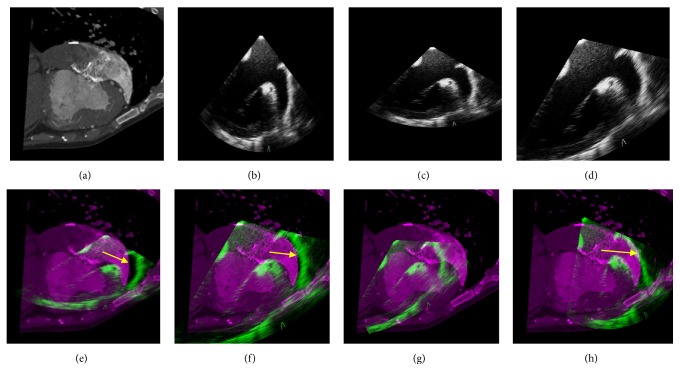
Registration process for a set of sections from an adult patient (Patient 12 in Table 1). (a) The original CT image. (b) The original TEE image. (c) The results of the basic registration. (d) The result of the final registration. (e) The fusion result based on the basic registration. (f) The fusion result based on the final registration. (g) The fusion result obtained from the comparison algorithm VMI. (h) The fusion result obtained from the comparison algorithm ICPMI. Note: the yellow arrows in (e), (f), and (h) indicate the alignment of the heart valves. CT, computed tomography; TEE, transesophageal electrocardiography; VMI and ICPMI, comparison algorithms.

Currently, there are very few studies on cardiac CT/MR-TEE registration, and each study focuses on different issues. D. S. Cho et al. [20] employed a landmark-based registration that was based on anatomical landmarks extracted using a custom-developed landmark extraction method. C. A. Linte et al. [21] studied the TEE-MR registration on porcine subjects, which used the mitral and aortic valve annuli as landmarks and required manual segmentation by professionals by using a custom-developed segmentation technique. P. Lang et al. [22] focused on the CT-TEE registration of the aortic root, which is only applicable to sections with complete aortic roots. None of the above algorithms can be directly implemented for the registration of our data. Therefore, we have only shown the target registration errors (TREs) of these algorithms in the original literatures and compared them with the maximum TRE of our algorithm in Table 1. F. Li et al. [23] performed CT-TEE registration according to the following steps: (1) semiautomatic segmentation was performed followed by execution of the initial registration based on ICP algorithm; (2) after the first-step alignment, the intensity threshold was used to extract the sample points, which were then applied to the registration based on the NMI. We employed this method (denoted by ICPMI) for our data as one of the comparison algorithms; the results are presented in Figures 5 and 6 and the indicators in Table 2. In addition, we used a method that optimized the NMI between the original CT and TEE images generated by the proposed basic registration as the comparison algorithm (denoted by VMI). The only difference between this comparison algorithm and the proposed algorithm is the use of the enhanced probability map; thus, our approach can be used to illustrate the performance of the enhanced probability map.

### 3.2. Visualization of the Results

The study was divided into two stages consisting of the basic and final registration. First, ROIs were manually segmented on both CT and TEE images using the grab-cut algorithm to locate the relevant valves. Based on those locations, we performed a simple and fast coarse registration of the original images to obtain the results of the basic registration. Afterward, the selected ROIs were enhanced and probability maps were generated. Finally, based on the transformation matrix obtained from the basic registration, we registered the enhanced probability map by optimizing the MI metric using Powell's optimizer.

Figures 5 and 6 show the process of registration for sets of sections from an infant patient (Patient 8 in Table 2) and an adult patient (Patient 12 in Table 2). Our algorithm did not produce any difference in the registration of infant and adult data. On comparing the fusion images of the basic and final registrations, we found that the basic registration was only a rough alignment of CT and TEE, while the final registration enabled the precise alignment of the valves (yellow arrows). Fusion images of the VMI (Figures 5(g) and 6(g)) were almost a complete failure of the registration performance, while the registration performance of ICPMI (Figures 5(h) and 6(h)) is also worse than the proposed algorithm. The differences between the VMI and the proposed method only lied in the enhanced probability map, which verified its great performance in registration. Our results revealed the exact alignment of the atria, ventricles, vessels, and valves in all patients.

### 3.3. Index Analysis

Index analysis was performed in order to evaluate quantitatively the performance of the proposed method and comparison algorithms. Dice coefficients [25] and registration errors are shown in Table 2. Final registration parameters were referenced to rigid registration methods [26]. First, ROI was obtained by manual segmentation. Each pixel within the ROI was transformed using both parameters of the proposed registration and the “bronze standard” registration [26]. And the root mean square (RMS) distance between these positions was calculated to yield a target registration error (TRE) (Table 2). Dice coefficients were obtained as follows:

(8) $$\begin{array}{c} Dice\;\;coefficient=\frac{2\left|A\cap B\right|}{\left|A+B\right|} \end{array}$$

with A and B representing the number of pixels in regions generated by transforming the artificial segmentation using the proposed method (or contrast algorithm) and the bronze standard registration, respectively, and *A*∩*B* represents the number of pixels in the overlapping area between them.

Table 1 shows the comparison of the maximum TREs between the existing methods and our method. Although the resolution and modality of the dataset used by each method are different, we can use this comparison to verify that our registration accuracy is within the acceptable range described in the current research on cardiac registration.

All measurements in Table 2 were based on an average over all 2D image slices for each patient. The average Dice coefficient of the proposed method reached 0.91 and the average TRE approached 1.2655 mm. These two indexes were notably better than those obtained by VMI from the 12 patients and ICPMI from the 11 patients besides Patient 9. In summary, the registration performance of our method was beyond the VMI and ICPMI in both infants and adult. The similarity of the enhanced probability maps in this paper worked better than the similarity of original images.

### 3.4. Performance Analysis

Our findings demonstrated the validity of probability mapping for the registration of cardiac CT and TEE images. Conventionally, infant data is more challenging to register compared to adult data due to the incomplete development of the heart and the smaller organs in infants. In our investigation, the registration performance of the proposed method was satisfying for both infant and adult data, which shows its good applicability to a variety of cardiac data.

Furthermore, the average time for optimizing MI of the probability map in our method was 7.45 s, which is substantially less than the average of 25.63 s for optimizing the original image under the same parameter conditions (Windows 7, 64-bit system, MATLAB 2015b operating environment). Therefore, the use of the probability map improved the observable accuracy of registration and reduced the registration time. Since CT interactive segmentation and feature point selection can be performed before surgery, interaction with TEE images is the only procedure necessary during the operation. This interaction involves simple markings with a mouse, on which professionals in this study spent a few seconds on each. Therefore, our method may be able to meet the requirements for real-time image guided surgery.

## 4. Conclusions

The imaging of congenital heart disease utilizes CMR, CT, TEE, and other technologies. Most existing image guidance surgery systems are based on traditional monomodal imaging and are therefore unable to provide comprehensive information about cardiac structures. In this study, we hypothesized that registering preoperative CT images to intraoperative TEE images can provide detailed navigation for congenital heart surgery. Compared to CT-MRI, MRI-US, and CT-PET registration of other organs in the human body, cardiac CT-TEE registration in infants is very challenging. Morphological differences caused by the deformity of heart tissues and heartbeat in addition to significant differences in gray levels between CT and TEE can complicate image registration. To address these issues, we proposed a large-deformation dynamic image registration method to meet the needs of surgical navigation in infants, young children, and adults with congenital heart disease. Our approach used valve positions to perform coarse registration, which was an enhanced probability map model for final registration and parameter transfer. These strategies were rapid and accurate. However, there were several limitations to our algorithm. First, basic registration required intervention during segmentation and feature point selection, which could have increased the workload of physicians and extended the duration of registration. Second, due to the difficulty of reconstructing TEE images, we implemented 2D-2D image registration without extending registration to a 3D space.

## Figures and Tables

**Algorithm 1:**
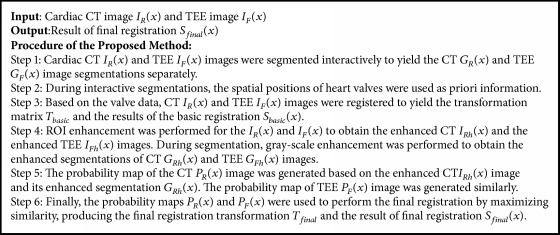
Procedure of the proposed method.

**Table 1:** Comparison of several registration algorithms on the maximum TRE.

Registration algorithm	D. S. Cho et al. [23]	C. A. Linte et al. [24]	P. Lang et al. [25]	Our method
TRE(mm)	5.1	4.8	5	2.3

TRE, target registration error.

**Table 2:** Registration indexes.

Patient	Average Dice coefficient			Average TRE (mm)		
	VPMMI	VMI	ICPMI	VPMMI	VMI	ICPMI
Patient 1	0.88	0.45	0.80	1.7290	9.5789	3.0710
Patient 2	0.89	0.80	0.82	1.2701	3.6162	1.8111
Patient 3	0.90	0.45	0.79	0.6013	11.5463	2.7112
Patient 4	0.95	0.95	0.84	1.1176	1.6780	8.0261
Patient 5	0.89	0.00	0.60	1.1179	12.3223	9.3880
Patient 6	0.87	0.04	0.88	2.2345	19.0837	4.9850
Patient 7	0.89	0.65	0.59	1.4118	7.5647	9.1564
Patient 8	0.91	0.32	0.89	0.8496	7.3520	0.8964
Patient 9	0.91	0.42	0.92	0.7337	5.7968	0.6936
Patient 10	0.95	0.83	0.85	0.6760	3.8212	2.9338
Patient 11 (adult)	0.90	0.58	0.69	1.7191	13.4195	6.6400
Patient 12 (adult)	0.92	0.62	0.86	1.7260	13.9903	2.2362
Average	0.91	0.51	0.79	1.2655	9.1475	4.3791

TRE, target registration error; VMI and ICPMI, the comparison algorithms; VPMMI, the proposed method (valves as a benchmark and probability map-based mutual information method)

## Data Availability

All test data in this research were obtained from Guangdong General Hospital in China. Sets of 22 standard TEE slices were collected from 12 patients with congenital heart disease using a PHILIPS S7-3t (Philips Medical Systems Technologies Ltd., Amsterdam‎, Netherlands) Pediatric with thermal index for soft tissue=0.1, mechanical index=0.3, frequency of the vibration=78 Hz, and 128 bpm. CT images were acquired from the same patients using a Siemens Somatom Definition (Siemens Healthcare, Forcheim, Germany) with voltage=100 kV, electric current=178 mA, multiplane reconstruction=0.8 mm, and Ctvidol=6.59. The image size of the TEE was 600 × 800 (pixel size=0.224 mm × 0.224 mm) and that of CT was 1024 × 1024 (pixel size=0.32 mm × 0.32 mm).
